# Image cytometry-based quantification protocol of human pulmonary arterial endothelial cells in lab-fabricated multichannel microfluidic devices

**DOI:** 10.1016/j.xpro.2025.104147

**Published:** 2025-10-21

**Authors:** Md Ibrahim, Sakib M. Moinuddin, Md Shahadat Hossain, Tri Nguyen, Tanoy Sarkar, Ahmed El-Shamy, Luca Cucullo, Tim Lahm, Eva Nozik, Marc A. Simon, Kurt R. Stenmark, Fakhrul Ahsan

**Affiliations:** 1 Department of Pharmaceutical and Biomedical Sciences, College of Pharmacy, California Northstate University, Elk Grove, CA 95757, USA; 2 Masters of Pharmaceutical Sciences Program, College of Graduate Studies, California Northstate University, Elk Grove, CA 95757, USA; 3 Veterans Affairs Northern California Health Care System, Martinez, CA 94553, USA; 4 Oakland University William Beaumont School of Medicine, 586 Pioneer Dr, Rochester, MI 48309, USA; 5 Division of Pulmonary, Critical Care and Sleep Medicine, National Jewish Health, Denver, CO 80206, USA; 6 Department of Pediatrics, University of Colorado Anschutz Medical Campus, Aurora, CO 80045, USA; 7 Division of Cardiology, Department of Medicine University of California, San Francisco, San Francisco, CA 94143, USA; 8 Oncovask Therapeutics LLC, Elk Grove, CA 95758, USA

**Keywords:** Cell Biology, Cell culture, High Throughput Screening, Microscopy, Molecular Biology, Biotechnology and bioengineering

## Abstract

Here, we present a protocol for automated quantification and viability analysis of pulmonary arterial cells (PACs) using a cellular image cytometer. We describe steps for obtaining PACs from the Pulmonary Hypertension Breakthrough Initiative, culturing and seeding them on microfluidic chips, and integrating chips into 6-well plates. Cells are stained with Hoechst 33342 and propidium iodide for live/dead analysis. Automated imaging and software-assisted quantification standardize workflows and broaden applications in pulmonary vascular research.

For complete details on the use and execution of this protocol, please refer to Al Hilal et al.^1^

## Highlights

• Steps for automated imaging and counting of cells on microfluidic chips
• Instructions for staining cells with Hoechst and propidium iodide dyes
• Procedures for positioning microfluidic devices in standard 6-well plate formats
• Guidance for setting up imaging protocols and software-assisted quantification

## Abstract (editor highlights)

Publisher’s note: Undertaking any experimental protocol requires adherence to local institutional guidelines for laboratory safety and ethics.

## Graphical abstract

**Figure:**
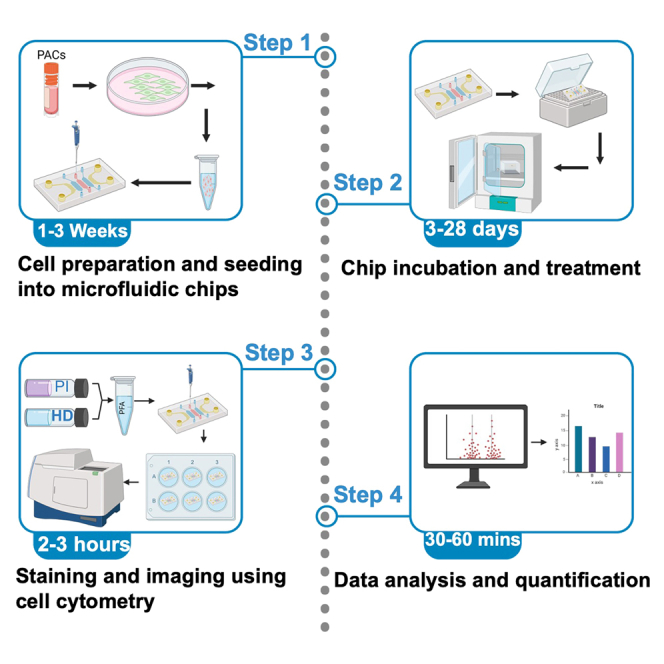

## Before you begin

This protocol builds upon our previous publication, which demonstrated the use of quantum dot trackers for cell counting and immunostaining of pulmonary arterial cells (PACs) within microfluidic devices.^1^

1. Obtain institutional approvals and ethical clearance for working with primary pulmonary arterial cells (PACs) derived from human subjects.
2. Prepare all necessary reagents and materials in advance, including supplements, growth media, and buffers, prior to receiving cell samples from the Pulmonary Hypertension Breakthrough Initiative (PHBI).
3. Ensure access to an image-based cellular cytometer, such as Celigo, Cytation, or Molecular Devices PICO. The cytometer must be capable of detecting Hoechst dye (excitation ∼350 nm, emission ∼461 nm) and Propidium Iodide (PI) (excitation ∼535 nm, emission ∼617 nm) for cell viability assays.
4. Refer to our prior protocol for detailed methodologies on handling PHBI-derived cell lines, including gelatin coating of culture vessels, cell expansion, and storage procedures.^2^
5. Obtain multicellular PAH-on-a-Chip platforms from MedLuidics, or fabricate them in-house using the chip design and fabrication methods described in our previous publications.^3^
6. Verify that your cellular image cytometer’s software supports the specific analysis tools required for your experiment, including cell viability and nuclear staining quantification.

### Innovation

This protocol enables high-throughput analysis of pulmonary arterial cells (PACs) in multichannel microfluidic chips adapted to a standard 6-well plate format. The design allows direct use with commercial image cytometers such as the ImageXpress Pico without the need for custom holders or chip modifications. By combining validated chip platforms with live/dead staining and automated quantification, the method improves reproducibility and scalability. The same platform also supports assays for cell cycle, apoptosis, mitochondrial function, and angiogenesis, extending its utility across diverse applications.

### Institutional permissions

Ensure necessary institutional ethics or protocol approval for cultivating, storing and maintaining primary pulmonary arterial cells (PACs) collected from human subjects. This includes obtaining permission from the Pulmonary Hypertension Breakthrough Initiative (PHBI) for the use of their cell specimen banks.

## Key resources table

**Table:** 

REAGENT or RESOURCE	SOURCE	IDENTIFIER
**Chemicals, peptides, and recombinant proteins**		

EBM-2 (Endothelial Cell Basal Medium-2)	Lonza	Cat# CC-3156
EGM 2 MV SingleQuots	Lonza	Cat# CC-4147
PBS (1×)	Thermo Fisher Scientific	Cat# 14040133
Trypsin-EDTA (0.25%), phenol red	Thermo Scientific	Cat# 25200072
Gelatin	Sigma-Aldrich	Cat# G9391
16% Formaldehyde (w/v), methanol-free	Thermo Fisher Scientific	Cat# 28906
Triton X-100	Thermo Fisher Scientific	Cat# A16046.AE
Propidium Iodide	Thermo Fisher Scientific	Cat# P1304MP
Hoechst 33342 solution (20 mM)	Thermo Fisher Scientific	Cat# 62249
Cell Banker 2 (100 mL) cryopreservation medium	Amsbio	Cat# 11914
Gibco Antibiotic-Antimycotic (100×)	Thermo Fisher Scientific	Cat# 15240062
Gibco Penicillin-Streptomycin (10,000 U/mL)	Thermo Fisher Scientific	Cat# 15140122
Photoresist SU-8 2100	Kayaku	Cat#Y1110750500L1GL
Photoresist SU-8 developer solution	Kayaku	Cat#Y0201004000L1PE
Acetone	VWR	Cat# 20067.290
99% Isopropyl alcohol-IPA	VWR	Cat# BDH2032-1GLP
Sylgard 184 Silicone Elastomer Kit	Avantor	Cat# 102092-312
Chlorotrimethylsilane, 98%	Thermo Scientific	Cat# 10214520
Deionized water	Thermo Scientific	Cat# 10977035
Phenol red sodium salt	Sigma-Aldrich	Cat# 14080-055
Poly-D-lysine (PDL) hydrobromide	Sigma-Aldrich	Cat# P7886
Rat collagen type I	Sigma-Aldrich	Cat# 08-115

**Experimental models: Cell lines**		

Idiopathic pulmonary arterial endothelial cells (PAH-ECs)	PHBI	N/A

**Software and algorithms**		

GraphPad Prism (version 10.2.3)	Dotmatics, MA, USA	N/A
CellReporterXpress Image Acquisition and Analysis Software, version 2.5	Molecular Devices	N/A
Leica Application Suite X (LAS X), version 5.3.0.	Leica Microsystems	N/A

**Other**		

Falcon tissue culture treated flasks (T-25)	Fisher Scientific	Cat# 10-126-10
Falcon tissue culture treated flasks (T-75)	Fisher Scientific	Cat# 13-680-65
PDMS Microfluidic PAH-on-Chip	Medluidics LLC	N/A
6-well clear TC-treated multiple well plates	Fisher Scientific	Cat# 07-200-83
Serological pipettes (5 mL)	Thermo Scientific	Cat# 12-567-602
Serological pipettes (10 mL)	Thermo Scientific	Cat# 12-567-603
Polypropylene conical centrifuge tubes (50 mL)	Fisher Scientific	Cat# 14-955-239
Polypropylene conical centrifuge tubes (15 mL)	Fisher Scientific	Cat# 14-959-70C
Cell freezing vial containers	Corning	Cat# 07-210-003
Freezing container	Thermo Scientific	Cat# 5100-0001
PES Filter, 0.22 μm, 75 mm diameter, sterile	CELLTREAT Scientific	Cat# 50-202-044
P10 Barrier pipette tips	Santa Cruz Biotechnology	Cat# sc-201721
Excel International TB SYR 1CC	Fisher Scientific	Cat# 22-841-001
Cleanroom wipes	Fisher Scientific	Cat# 17-444-001
Luer Lock 10 mL sterile syringes	VWR	Cat# 53548-023
Syringe Filter, PVDF, 0.22 μm	Fisher Scientific	Cat# NC0992876
Silicon Wafer (100 mm, 4″)	University Wafer	Cat# 452
Single Wafer Carrier Box, 4″	MTI Corporation	Cat# SP5-S4
Wafer handling tweezers	Excelta	Cat# 490L-SA-PI
5″ Glass Petri dish	Cole Parmer	Cat# EW-34551-05
Whatman Grade 2 qualitative filter paper	TISCH Scientific	Cat# 1002-090
Tri-cornered polypropylene beakers	Fisher Scientific	Cat# 14-955-111A
Glass stirring rods	VWR	Cat# 59060-105
Cutting Matt	Newark	Cat# PKN6003
Scalpel Handle-straight	Excelta	Part # 177-SE
Scalpel blades #10 (sterile)	Excelta	Part # 181-10
Plastic Petri dishes with clear lid	Fisher Scientific	Cat# FB0875714
Integra Miltex Standard 1 mm Biopsy Punches	Fisher Scientific	Cat# 12-460-401
Integra Miltex Standard 4 mm Biopsy Punches	Fisher Scientific	Cat# 12-460-409
Style 5 Ultra-fine pointed tweezers	Excelta	Cat# 5-SA
Microscope cover glass	Fisher Scientific	Cat# 12-545-AP
Tissue Path Superfrost Plus Gold Slides	Fisher Scientific	Cat# 22-035813
Benchmark SureTherm 180 CO_2_ incubator	Benchmark	H3565-180
Class II biological safety cabinet	LABCONCO	Cat# 302519100
Pump aspirator	Welch	Cat# 2534B
Water bath (Precision 183)	Precision Scientific	Cat# 66551-27
Disposable glass pipets, 9 inch	Fisher Scientific	Cat# 50-136-7739
Biological microscope	Proway Optics & Electronics Co.	XSZ-PW107
Benchtop centrifuge	Eppendorf AG	5805F
Molecular device Pico	Molecular Devices, LLC	N/A
Spin Coater	Laurell Technologies Corp.	Cat# WS-650-23
LEICA DMi8 manual microscope	Leica Microsystems	DMi8
Wafer Alignment Tool	Laurell Technologies Corp.	N/A
Solid state heat/cool cold plate	Teca	Ref# AHP-1200CPV
UV-LED Masking System: UV-KUB 2	KLOE	N/A
High power expanded plasma cleaner	Harrick Plasma	Cat# PDC-001-HP
Benchtop decontamination chambers	Air Science	Cat# WBB2173526
Vacuum desiccators	Fisher Scientific	N/A
Isotemp 500 Series Economy Lab Ovens	Fisher Scientific	Cat# 13246516GAQ
Bright-Line Hemacytometer	MilliporeSigma	Cat# Z359629

## Materials and equipment

**Table:** PDMS liquid mold

Reagent	Final concentration	Amount
SYLGARD 184 elastomer base	N/A	40 g
SYLGARD 184	N/A	4 g
Total	10:1 weight ratio	44 g

Prepare fresh before use.

**Table:** PDL solution

Reagent	Final concentration	Amount
PDL powder	N/A	10 mg PDL
Sterile distilled water	N/A	1 mL
Total	10 mg/mL	1 mL

Prepare fresh before use.

**Table:** 0.2% gelatin solution

Reagent	Final concentration	Amount
Gelatin	0.2%	0.2 gm
ddH_2_O	N/A	100 mL
Total	N/A	100 mL

Store at 2°C–8°C for up to 4 weeks.

**Table:** Pulmonary endothelial cell (ECs) medium

Reagent	Final concentration	Amount
EBM-2	N/A	470 mL
EGM 2 MV SingleQuots (contains bellow components)	–	–
FBS	5.0%	25.0 mL
Hydrocortisone	0.04%	0.2 mL
hFGF-B	0.4%	2.0 mL
VEGF	0.1%	0.5 mL
R3-IGF-1	0.1%	0.5 mL
Ascorbic Acid	0.1%	0.5 mL
hEGF	0.1%	0.5 mL
GA-1000	0.1%	0.5 mL
**Total**	**N/A**	**500 mL**

Store at 2°C–4°C up to 2 months.

## Step-by-step method details

### Obtaining pulmonary arterial cells from the PHBI

**Timing: 2–3 weeks**

Researchers from academic and non-profit organizations in the USA can apply to the Pulmonary Hypertension Breakthrough Initiative (PHBI) for access to cells obtained from patients with PAH.

1. Create an account on the PHBI electronic Tissue Utilization Committee (eTUC) application system at http://phbi.org/index.do.
2. Pay the associated fee for the sample.
3. Receive the replicating cells from PHBI in a T-25 flask.
4. Replace the existing medium with 5 mL of fresh, pre-warmed (37°C) appropriate cell culture medium.
5. Incubate the T-25 flask in an incubator at 37°C with 5% CO_2_ until the cells reach 60%–70% confluency.

### Coating of plates for culturing and expanding pulmonary arterial cells

**Timing: 7–8 h**

6. Preparation of 0.2% Gelatin Solution.
  a. Weigh 0.2 g of gelatin powder.
  b. Transfer the powder into a sterile volumetric flask and add sterile distilled water to a final volume of 100 mL.
  c. Vortex thoroughly until the gelatin dissolves completely and the solution appears clear.
  d. Autoclave the solution at 121°C under 15 psi for 35 min to ensure sterility.

***Note:*** The prepared 0.2% gelatin solution, as well as gelatin-coated plates or flasks, can be stored at 2°C to 8°C for up to 4 weeks, provided they are sealed properly to prevent contamination. Discard any solution or coated surface showing visible cracks, discoloration, or microbial growth.

7. Coating Procedure.
  a. To coat the culture surfaces, transfer 500 μL of the 0.2% gelatin solution into each well of a 6-well or 24-well plate. For larger culture vessels, add 1.5 mL to T-25 flasks and 5 mL to T-75 flasks.
  b. Ensure the solution evenly covers the entire surface. Close the lids and incubate the plates or flasks at room temperature (22°C–25°C) for 2 h.
  c. After incubation, aspirate the solution, then air-dry flasks in a biosafety cabinet for ≥4 h.
  d. Before seeding cells, verify that no residual liquid remains and that the surface is completely dry.

### Culturing, expansion, and cryopreservation of pulmonary arterial hypertension cells

**Timing: 1–3 weeks**

8. Monitor cell morphology and confluency daily under an inverted microscope. Proceed when cells reach approximately 80%–90% confluency.
9. Pre-warm the appropriate culture medium, 1× PBS, and 0.25% Trypsin-EDTA by placing them in a 37°C water bath for at least 15–20 min prior to use.
10. Examine and document the morphology of the cells to confirm they are healthy (e.g., elongated, spindle-shaped for endothelial cells) and free of contamination (e.g., bacterial or fungal).
11. Aspirate the spent medium from the T-25 flask under sterile conditions in the biosafety cabinet.
12. Gently rinse the cell monolayer with 5 mL of sterile 1× PBS to remove residual serum that may inhibit trypsin activity. Aspirate the PBS completely.
13. Add 1.5 mL of 0.25% Trypsin-EDTA to the flask, ensuring the entire surface is covered. Incubate at 37°C for approximately 10 min.

***Note:*** During trypsinization, cells will begin to detach and round up. Observe the process under the microscope. PAH cells may be more adherent and require a slightly longer incubation time. Do not exceed 15 min to avoid damaging cells.

14. Once cells are detached, immediately neutralize the trypsin by adding an equal or greater volume of complete growth medium containing serum (e.g., 1.5–3 mL).
15. Tap the flask gently to dislodge residual attached cells. Pipette the suspension up and down 10–15 times to achieve a single-cell suspension.
16. Transfer the cell suspension into a sterile 15 mL conical (Falcon) tube.
17. Centrifuge the tube at 413 × g (≈1,200 rpm), 5 min at 4°C, with acceleration level 3 and deceleration level 0 (gentle start, free stop).
18. Return the tube to the biosafety cabinet and carefully aspirate the supernatant without disturbing the cell pellet.
19. Resuspend the pellet in 2–3 mL of pre-warmed complete growth medium by gentle pipetting.
20. Count the cells using a hemocytometer or an automated cell counter to determine viability and cell number.
21. Seed approximately 1 × 10^6^ cells into a T-75 flask containing 12 mL of complete culture medium. Incubate at 37°C in a humidified incubator with 5% CO_2_ and 95% air for further expansion.
22. Monitor daily for cell growth. When the culture reaches ∼60%–70% confluency (typically within 3–5 days), repeat the passaging steps (Steps 10–20) for further expansion or experimental use.
23. For cryopreservation, prepare cryovials by resuspending approximately 5 × 10^5^ cells in 1.5 mL of Cell Banker 2 or another validated cryopreservation medium. Freeze cells ideally at early passages for optimal recovery and viability.

### Microfluidic PDMS chips: Design, mold preparation, fabrication, and sterilization

**Timing: 2–3 days**

For detailed fabrication methods, please refer to our published paper.^3^ This summary offers approximate estimates of the time and resources required.

**Figure 1:**
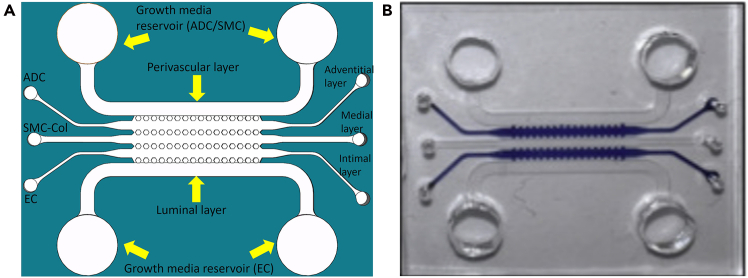
Multichannel PAH microfluidic chip design and fabricated device in use (A) 3D CAD schematic of the multichannel pulmonary arterial hypertension (PAH) microfluidic chip. The chip contains five microchannels designed to simulate pulmonary artery architecture, including three cellular layers: adventitial cells (ADCs) in the outer adventitial layer, smooth muscle cells (SMCs) in the medial layer, and endothelial cells (ECs) in the inner intimal layer. The device features separate media reservoirs for SMCs and ADCs in the perivascular compartment and for ECs in the luminal compartment. (B) Photograph of the fabricated microfluidic chip in use.

**Figure 2:**
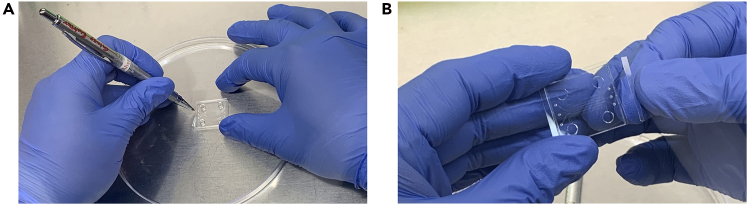
Removal of excess glass surrounding the plasma-bonded microfluidic chip using a diamond cutter (A) Demonstration of the diamond cutter held like a pen to precisely score the glass; the microfluidic chip is positioned in a clean, sterile Petri dish. (B) Example showing removal of glass slide edges with the diamond cutter.

24. Design the chip in CAD software (e.g., SolidWorks) featuring five channels with hexagonal pillar structures (Figure 1). Chip design services, such as Parallel Fluidics, can be utilized if necessary.
25. Submit the finalized 3D chip design to a photomask manufacturer (e.g., Artnetpro Inc.). A typical photomask order, including file conversion and an 11″×14″ 20K mask with ground shipment, costs approximately $146.50.
26. Clean a silicon wafer thoroughly with compressed air and spin-coat SU-8 2100 photoresist to achieve a thickness of approximately 150 μm.
27. Perform a soft bake of the coated wafer at the recommended temperature for 1 h.
28. Align the photomask directly onto the photoresist-coated side of the wafer, ensuring firm and even contact. Expose the wafer to UV light through the photomask using a UV-KUB 2 exposure system for approximately 10 min.
29. Conduct a second soft bake on the UV-exposed wafer for 2.5 h.
30. Develop the wafer by immersing it in an SU-8 developer solution for about 8 min until clear structural patterns emerge. Rinse thoroughly with isopropanol for at least 30 s to remove residual developer solution.
31. Carefully dry both wafer sides with compressed air until completely dry and free of visible residues.
32. Inspect the wafer microscopically, ensuring the mold features sharp edges, linear channels, and precisely defined channel structures without undesired curvature.
33. Create a master mold by placing the wafer into a sterile petri dish, secure the wafer corners with tape, and seal with the lid to avoid contamination for future use.
34. Prepare PDMS by mixing PDMS base and curing agent at a 10:1 ratio. Degas the PDMS mixture under vacuum and carefully pour onto the wafer master mold.
35. Cure the PDMS mixture on the master mold at 80°C for 2 h. After cooling, carefully peel off the cured PDMS from the mold.
36. Cut the PDMS into individual microfluidic chips and punch inlet and outlet ports with a biopsy punch.
37. Plasma-bond the PDMS chips onto sterile glass slides. Trim excess edges using a diamond cutter (Figure 2).
38. Place the bonded PDMS-glass chips into a 65°C incubator for 2 h, followed by an additional curing period at 80°C for 1 h.
39. Autoclave the completed chips at 121°C, 15 psi for 20–30 min if sterilization is required. Allow chips to cool and dry in a sterile biosafety cabinet.
40. Store sterilized chips at room temperature (20°C–25°C) in sealed sterile Petri dishes for up to 2 weeks. For longer storage, chips may be kept at 4°C for up to 4 weeks. If chips are stored longer than 1 week, re-treat with plasma cleaning before cell seeding to restore hydrophilicity.

**Figure 3:**
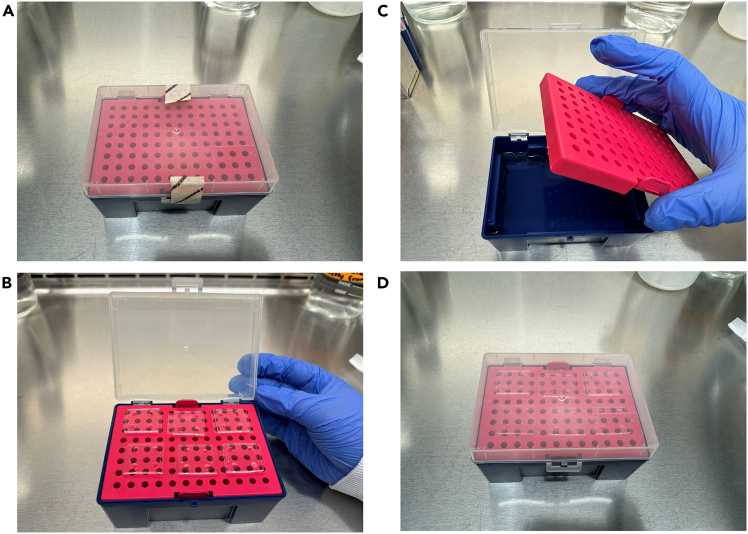
Stepwise illustration of a custom humidity chamber for incubating cell-seeded microfluidic chips (A) Sterilized pipette box. (B) Lower compartment filled with sterile, autoclaved water. (C) Microfluidic chips placed inside, prepared for poly-D-lysine (PDL) coating or cell seeding. (D) Box closed and incubated at 37°C with 5% CO_2_.

**Figure 4:**
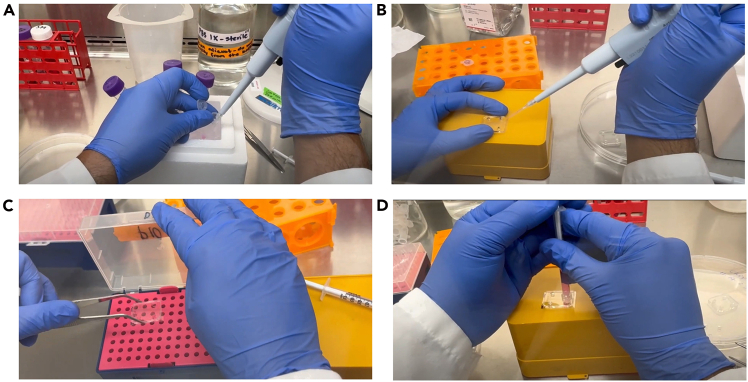
Cell seeding process in the microfluidic chip (A) Draw 10 μL of cell suspension using a P10 pipette. (B) Introduce the suspension into the targeted channel. (C) Incubate the chip in a humidified chamber (pipette-tip box) at 37°C for 4–5 h. (D) After incubation, use a 1 mL syringe to add culture medium through the reservoirs, then return the chip to the incubator.

**Figure 5:**
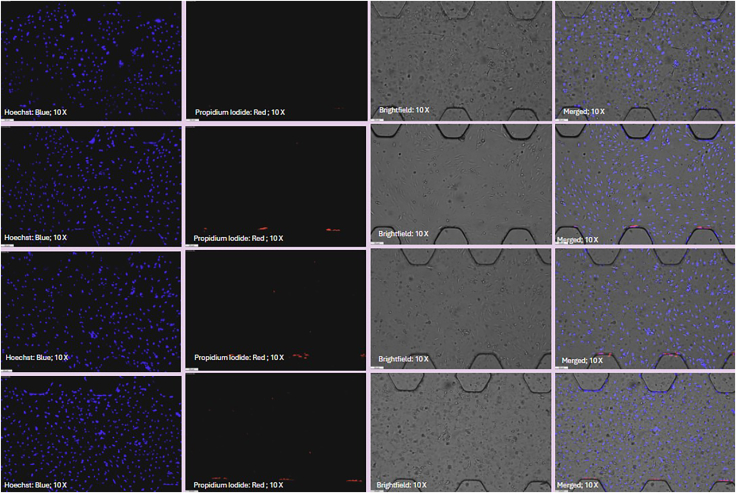
Pulmonary artery endothelial cells (PAH-ECs) from PAH-afflicted human arteries seeded in the microfluidic chip and stained with Hoechst dye (HD) and propidium iodide (PI) Immunofluorescence imaging at 24, 48, 72, and 96 h shows morphological changes and increasing cell density over time.

**Figure 6:**
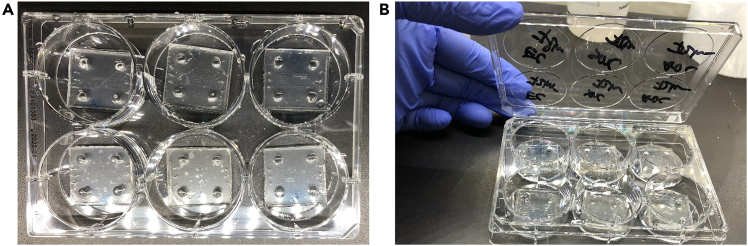
Preparation of a six-well plate containing microfluidic chips (A) Chips placed into a clean, sterile six-well plate. (B) Each well clearly labeled to avoid mix-up between experimental groups.

**Figure 7:**
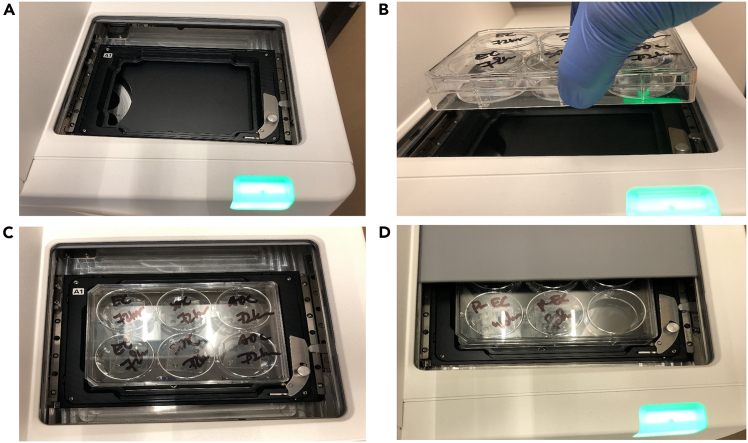
Placement of a six-well plate with microfluidic chips into the Molecular Devices PICO imaging system (A) Turn on the system and press the green chamber button to open the imaging chamber. (B) Insert the plate containing chips. (C) Align channels properly for imaging. (D) Close the chamber securely.

**Figure 8:**
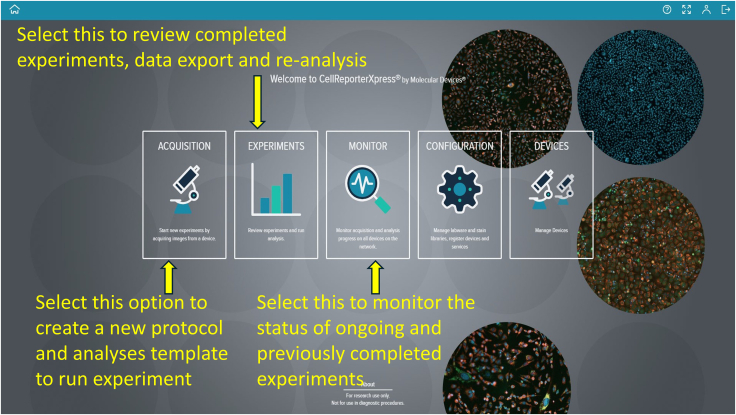
Landing page of the CellReporterXpress software from Molecular Devices, LLC, showing options for creating protocols, running experiments, performing analyses, and monitoring progress

**Figure 9:**
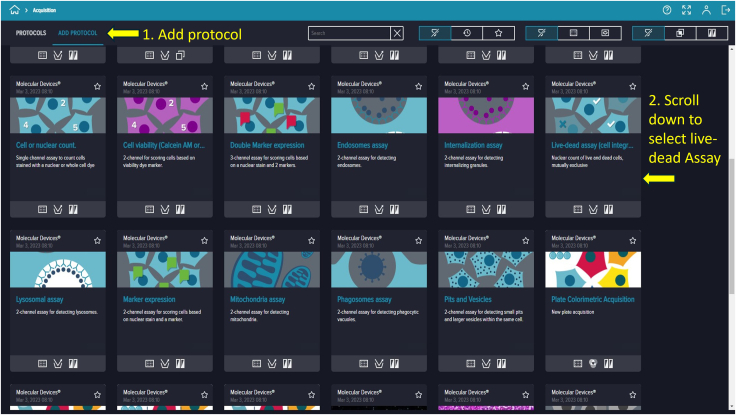
Adding a protocol Click *Add Protocol* to load a template for experiments. Use the search bar or mouse to locate and select the desired template.

**Figure 10:**
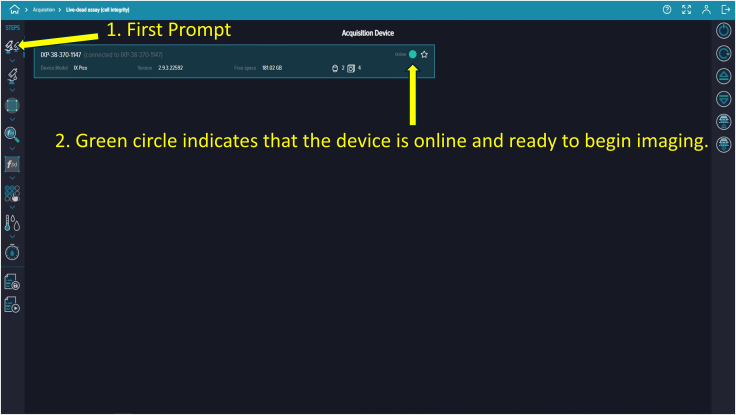
“Prompts” refer to functional steps listed vertically under the *STEPS* section Selecting each prompt allows customization of built-in protocols.

**Figure 11:**
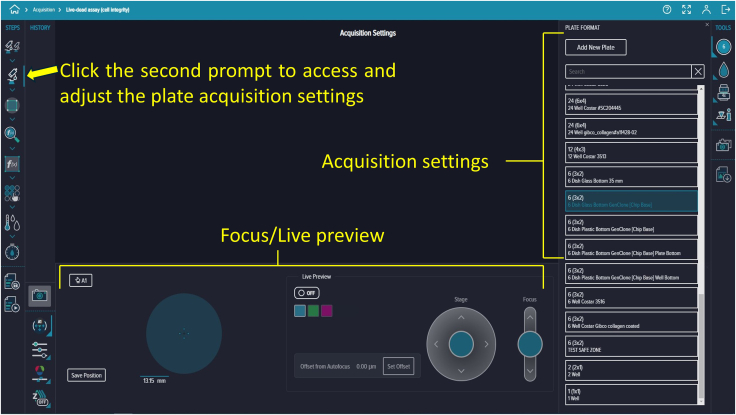
Plate acquisition settings Imaging parameters are adjusted in the panel on the right, with autofocus and live preview options at the bottom.

**Figure 12:**
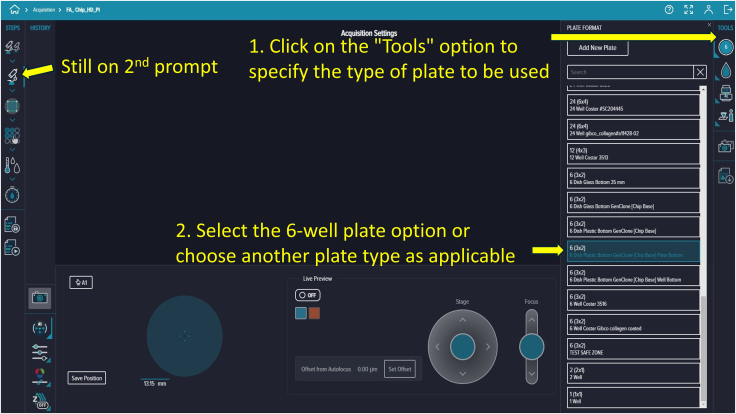
Plate selection during setup Use the *Tools* option on the right to specify plate type. Select the 6-well plate option or another type as appropriate.

**Figure 13:**
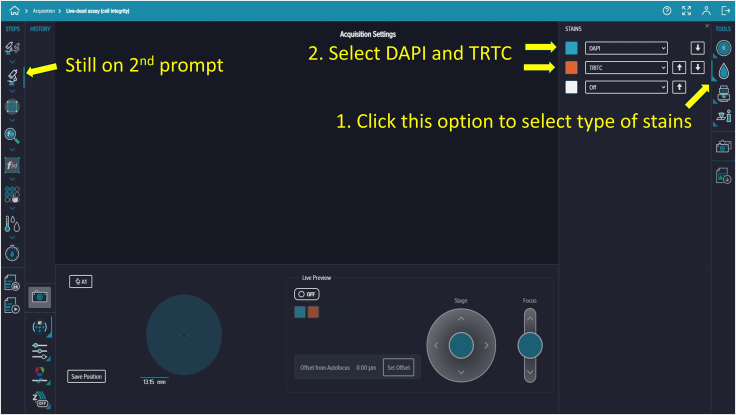
Stain selection Use the second *Tools* option on the right to specify staining channels. Select DAPI and TRITC as required.

**Figure 14:**
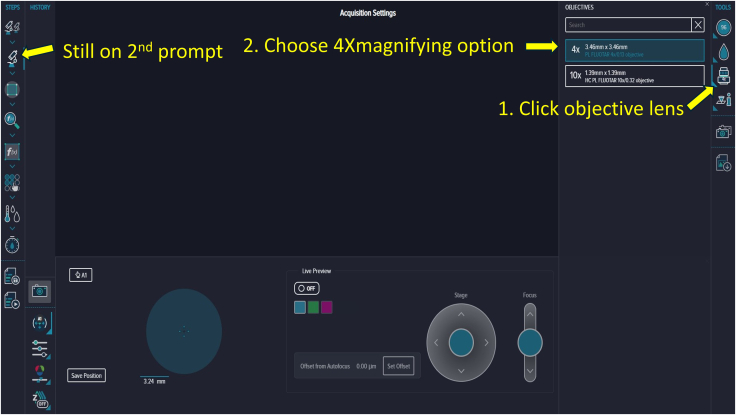
Objective lens selection Use the third *Tools* option on the right to specify magnification; a 4× lens was used here.

**Figure 15:**
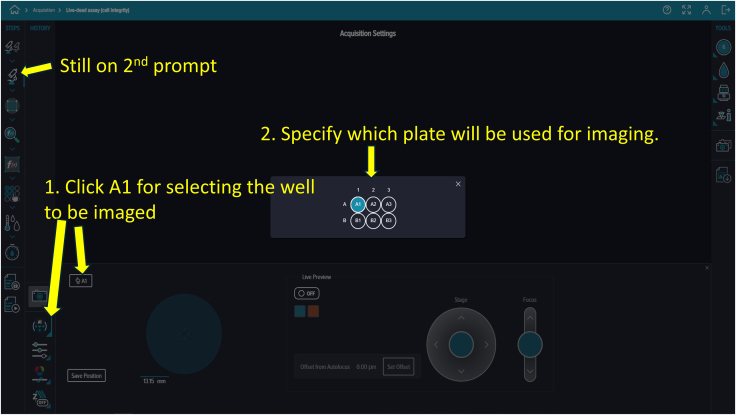
Well selection This option allows the user to choose which wells will be imaged.

**Figure 16:**
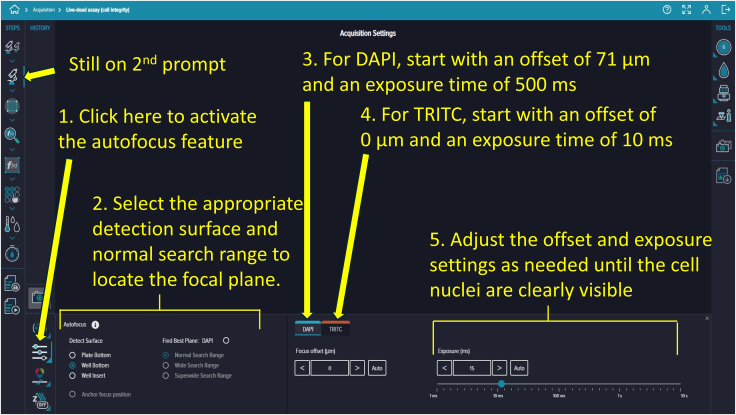
Autofocus procedure Autofocus is performed using the plate bottom and a defined search range. Offset and exposure settings are optimized (e.g., DAPI: +71 μm, 500 μs; TRITC: 0 μm, 10 μs) to visualize nuclei.

**Figure 17:**
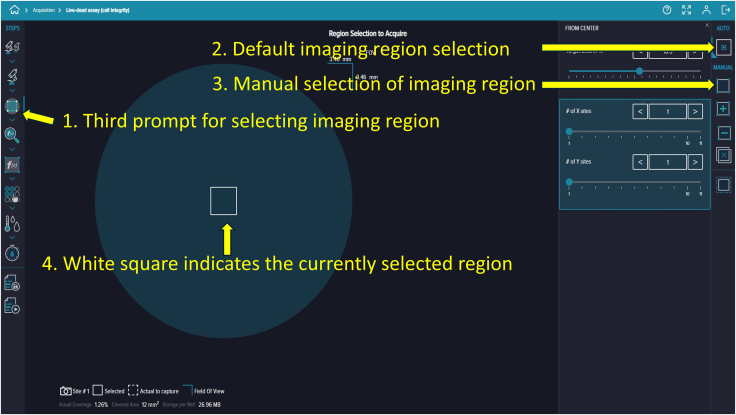
Imaging region selection Default or manual modes can be used, with the selected region outlined by a white square.

**Figure 18:**
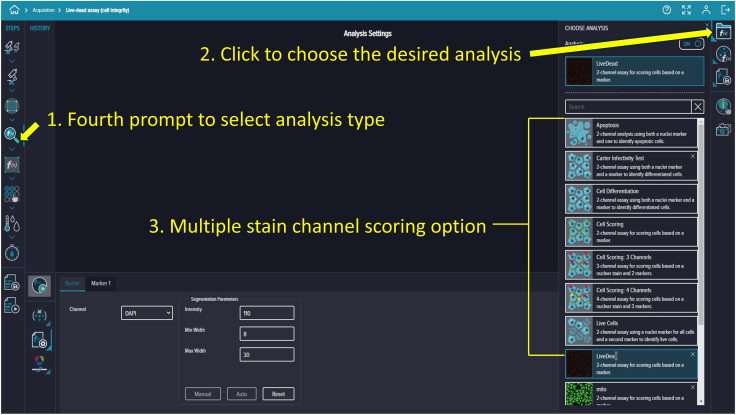
Analysis type selection Interface for choosing analysis type and stain channel scoring options.

**Figure 19:**
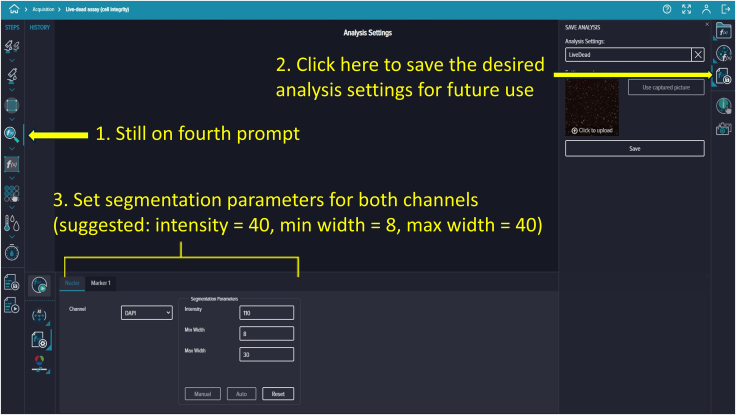
Analysis setup interface, including prompts, save option, and initial segmentation parameters for both channels

**Figure 20:**
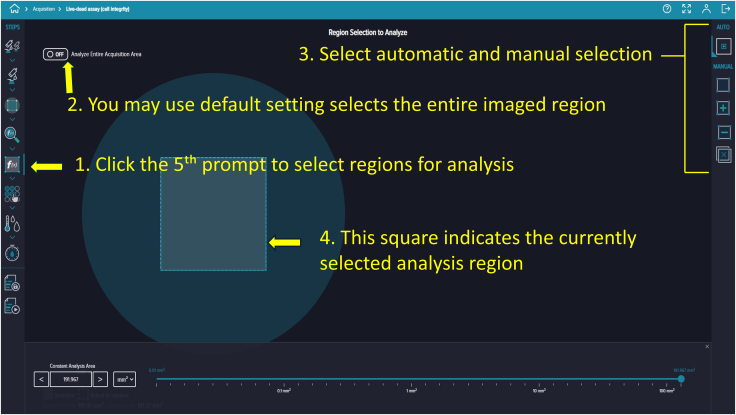
Region selection for analysis Options include default, manual, or automatic selection, with the chosen area visually indicated.

**Figure 21:**
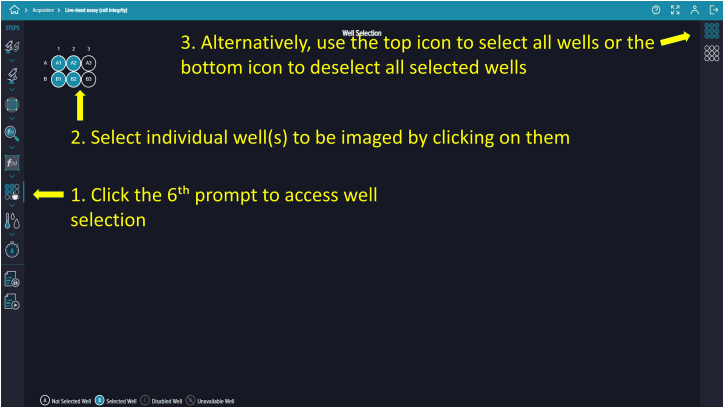
Well selection interface for imaging and analysis, allowing manual or bulk selection of wells

**Figure 22:**
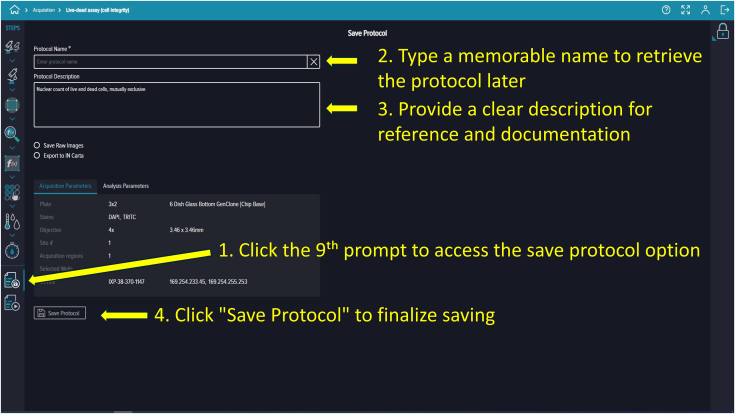
Saving the protocol Fields for protocol name and description are provided, along with confirmation of stored settings.

**Figure 23:**
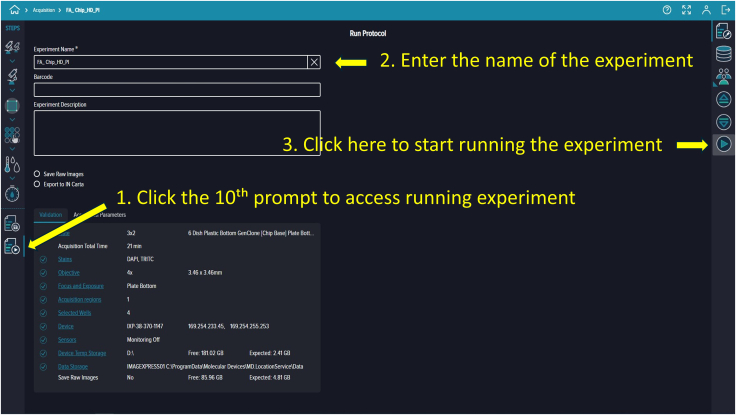
Running the experiment Users enter experiment details and execute the protocol.

**Figure 24:**
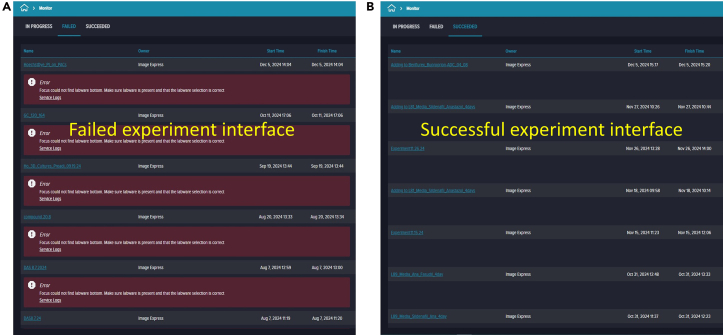
Examples of failed and successful experiment runs (A) failed run with focus errors due to incorrect labware selection. (B) successfully completed run with timestamps.

### Poly-D-lysine coating, cleaning, and restoring hydrophilicity of chips

**Timing: 1–3 days**

41. Prepare a Poly-D-Lysine (PDL) solution and sterilize it by passing through a 0.22 μm filter to remove insoluble particles.
42. Inside a sterile cell culture hood, place microfluidic chips on a flat, clean surface such as an autoclaved pipette box containing sterile water to create a humidified chamber.
43. Using a P10 micropipette, gently aspirate 10 μL of PDL solution and slowly inject it into the inlet of the intimal channel until it reaches the outlet. Hexagonal pillars within the chip will assist surface tension in preventing leakage.
44. Carefully inspect for any leakage of PDL solution into adjacent channels. If no leakage is observed, repeat the procedure for the adventitial channel.
45. Inject PDL solution into the medial channel until approximately one-third of the channel length is filled. Leave around 5 μL solution at the inlet, then use a micropipette to aspirate air from the outlet to ensure complete filling. Discard chips exhibiting leakage after marking the leaking channel.
46. Using a sterile P200 micropipette, add PDL solution into the reservoir inlet and gently pipette up and down to ensure the solution evenly distributes across the chip through hydraulic pressure.
47. Incubate chips under sterile conditions within the humidifying chamber at 37°C for 2 h or 10–12 h at 4°C.
48. After incubation, carefully aspirate any residual PDL solution from the chips using a sterile glass aspirator inside a cell culture hood.
49. With a sterile P200 micropipette, thoroughly rinse each chip’s reservoir channels three times by adding and pipetting 100 μL of sterile deionized water.
50. Place the rinsed chips into a sterile 150 mm glass Petri dish and incubate at 85°C for 12–24 h to restore hydrophilicity, enhancing subsequent cell adhesion and growth. If chips have been stored for more than 1 week, perform plasma cleaning prior to this step to ensure optimal surface activation.
51. Following incubation, further sterilize the chips by exposing them to UV sterilization chamber for 1 h.
52. Gently remove any remaining dust particles from the chip surfaces by wiping lightly with an alcohol-sprayed chem wipe.
53. The microfluidic PAH-chips are now ready for cell seeding.

### Cell seeding into microfluidic chips, nourishment of seeded cells, incubation, and monitoring

**Timing: 5–7 days**

54. Split available pulmonary arterial endothelial cells (PAH-ECs) according to standard cell culture protocols for cells derived from pulmonary arteries.
55. Prepare a cell suspension at a concentration of 10 × 10^6^ cells/mL. Typically, add 5 × 10^5^ PAH-ECs into 50 μL of suitable cell culture medium.
56. Use a clean, autoclaved pipette box containing sterile water to create a custom humidifying chamber.
57. Inside a sterile cell culture hood, place UV-sterilized microfluidic PAH-chips onto the surface of a clean autoclaved pipette box (Figure 3).
58. Using a P10 micropipette, gently resuspend the cell suspension. Aspirate 10 μL and carefully insert the pipette tip into the inlet of the chip’s middle channel. Slowly dispense the suspension until it flows to the outlet of the designated channel (Figure 4).
59. Incubate the chip at 37°C with 5% CO_2_ for at least 6 h to allow cells to attach and settle within the designated channel. Observe microscopically to confirm proper cell attachment and layering.
60. After the initial incubation, carefully add sufficient cell culture medium into the chip channels and reservoirs to maintain cell nourishment.
61. Place the seeded chips into the custom-made humidifying chamber and incubate at 37°C with 5% CO_2_.
62. Stain the chips and acquire images every 24 h over a 3–7-day period as required by your experimental protocol, following the staining method described below (Figure 5).

### Optional daily medium replacement

**Timing: 30 min**

63. Depending on your experimental conditions, replace old medium from the chip channels and reservoirs as needed. Using a P10 micropipette, remove old medium from channel outlets, and replenish with 6–7 μL fresh growth medium per channel using a P200 micropipette from the reservoirs.

### Staining pulmonary arterial cells with nuclear dye

**Timing: 2–3 h**

64. To prepare 2 mL of staining solution, dilute appropriate volumes of stock solutions of PI and Hoechst dye into PBS to achieve final concentrations of 1 μg/mL PI and 5 μg/mL Hoechst.
65. Using a micropipette, add 500 μL of the mixed dye solution into the microfluidic device through the designated channels.
66. Ensure the dye evenly covers all cells. Wrap the device in aluminum foil to protect from light.
67. Incubate the chip at 37°C with 5% CO_2_ for 30 min.
68. Depending on the background signal, gently wash the chip with 500 μL PBS to remove excess dye.

### Preparing microfluidic devices with Molecular Devices PICO

**Timing: 30 min**

69. Preparation of Six-Well Plate and Microfluidic Devices (Figure 6).
  a. Obtain a sterile or clean six-well plate.
  b. Place the microfluidic chips into the wells of the plate.
  c. Close the plate lid to avoid contamination.
  d. Clearly label each chip on the lid.
70. Preparation of Molecular Device PICO^4^ for Imaging (Figure 7).
  a. Turn on the Molecular Device PICO using the power button if not already on.
  b. Press the illuminated green chamber button to open the imaging chamber.
  c. Place the six-well plate into the imaging chamber, ensuring proper alignment.
  d. Press the chamber button again to close the chamber securely.

### Creating and starting the imaging protocol

**Timing: 30 min**

71. Access the computer with the complementary software “CellReporterXpress.” A landing page displaying five options—Acquisition, Experiments, Monitor, Configuration, and Devices—will appear (Figure 8).
72. Click “Acquisition” to view available protocol templates.
73. On the protocol page, click “Add Protocol” to locate and proceed with “Live-dead assay (cell integration).” While this template fits this experiment, many other templates are available for different assays (Figure 9).
74. Select the first prompt, indicated by a dual microscope icon on the top left corner under “STEPS” and verify the device is online, marked by a green circle (Figure 10).

***Note:*** “Prompts” refer to functional steps listed vertically under the “STEPS” section on the screen, numbered from top to bottom.

75. Based on your experimental requirements, adjust the existing protocol. Click the second prompt to display the Acquisition Settings panel on the right side of the screen, which includes the focus setting and a live preview option at the bottom (Figure 11).
76. Click the first icon in the top-right corner of the Acquisition Settings to select the 6-well plate template (Figure 12).

***Note:*** Plate settings from any vendor can be imported into the software by following your machine’s manual or instructions.

77. Click the second icon in Acquisition Settings to choose your stains. For example, we selected DAPI (Hoechst; Ex/Em = 350/461 nm) and TRITC (Propidium Iodide; Ex/Em = 535/617 nm) (Figure 13).
78. Click the third icon in Acquisition Settings to select the desired objective lens. We used a 4× objective (Figure 14).
79. In the focus settings and live preview at the bottom, click “A1” to specify which wells contain your microfluidic device (Figure 15).
80. To adjust focus, click the “Focus” button (three dots) in the focus settings and live preview section. We selected the plate-bottom-based focus method and initially used the DAPI channel to achieve optimal focus (Figure 16).
81. Fine-tune the focus for both DAPI and TRITC channels using autofocus or manually adjusting the focus offset and exposure settings. Click the camera icon to take a snapshot and evaluate image quality. Example settings: DAPI (+71 μm offset, 500 μs exposure), TRITC (0 μm offset, 10 μs exposure) (Figure 16).

### Defining imaging region and analysis settings

**Timing: 15 min**

82. To select the image acquisition area of your microfluidic device, click the third prompt. You can image either the entire well or just the area where cells are seeded on the chip.
83. Customize the square-shaped outline to select the region of interest manually or automatically using the settings provided on the right side (Figure 17).
84. To add analysis to the protocol, click the fourth prompt. This will open the “Choose Analysis” options on the right side. Select any suitable 2-channel cell-scoring analysis (Figure 18).
85. We selected DAPI as the first channel for marking nuclei (live and dead cells) and TRITC as the second channel for PI labeling to detect dead cells. Initial intensity settings of 40, minimum width of 8, and maximum width of 40 are reasonable. Adjust these values as needed during reanalysis to prevent nonspecific detection. You can save and name these settings for future analyses (Figure 19).
86. To add a captured image region for automatic analysis, click the fifth prompt and select the region as described in Step 96 (Figure 20).

### Well selection, temperature control, saving, and running protocol

**Timing: 15 min**

87. Specify wells to image and analyze by clicking the sixth prompt (Figure 21).

***Optional:*** For temperature-controlled, time-dependent acquisitions, use the seventh prompt. We did not use this.

***Optional:*** Use the default system settings in the eighth prompt for the imaging direction/order of wells unless a custom sequence is required. We used the default settings.

88. To save your protocol for future use, click the ninth prompt. Provide an identifiable assay name and description (e.g., “Live-Dead Microfluidic Assay”) (Figure 22).
89. Prompt ten runs the saved protocol. Enter your experiment name and details, then click the “Play” button on the right side of the software screen to start (Figure 23).
90. Once you click “Run,” the software navigates to the “Monitor Page.” The run status can appear as In Progress (ongoing imaging and analysis), Failed (requires troubleshooting), or Succeeded (imaging and analysis completed) (Figure 24).
91. When the run finishes and shows “Succeeded,” return to the landing page by clicking the house icon at the top-left corner, then click “Experiments” to view captured images and analyzed data.

### Data reanalysis, exporting, and presentation

**Timing: 45 min**

Steps 84–106 include image capture and analysis. However, if nonspecific or unusual cell counts are detected during evaluation, reanalysis (adjusting intensity thresholds, min/max width, etc.) may be necessary.

**Figure 25:**
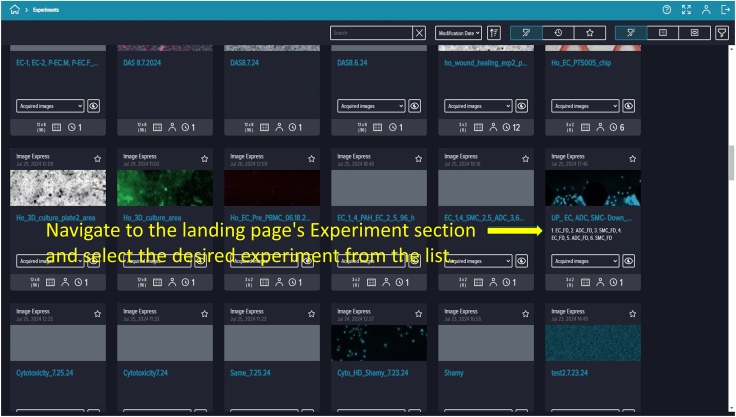
Locating completed experiments Navigate to the *Experiments* section of the landing page and select the desired experiment.

**Figure 26:**
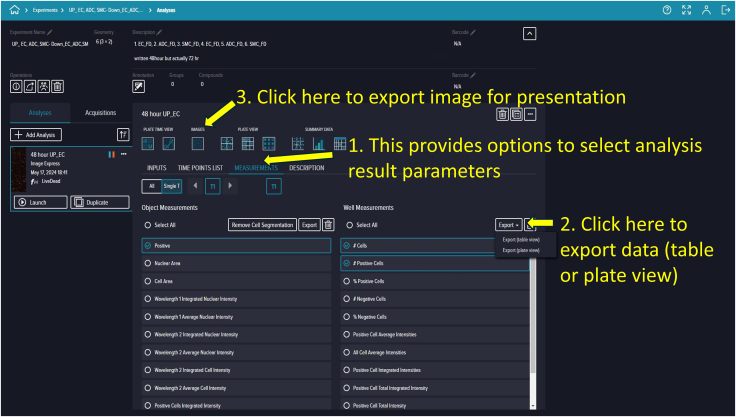
Analysis interface showing parameter selection and export options Users can select metrics (e.g., total cells, PI-positive cells) and export data. Images can also be extracted for validation or presentation.

**Figure 27:**
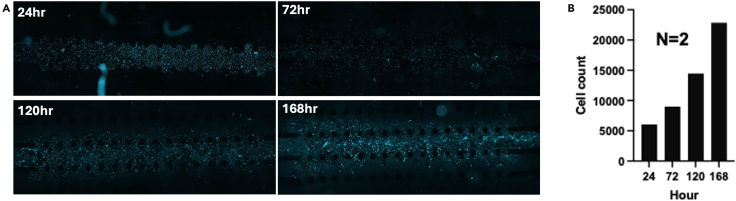
Representative fluorescence images of cell distribution and density at 24, 72, 120, and 168 h (A) Images show progressive increases in cell accumulation and organization. (B) Quantitative analysis can be performed as needed.

92. To reanalyze data, navigate to the Cell Reporter Xpress landing page and click “Experiments.” Your recent experiment should appear; if not, locate it using the provided search bar. Click on your experiment (Figure 25).
93. Opening your experiment reveals two sections: “Analyses” and “Acquisitions.” To review data, click “Analyses” then “Measurements.” Select either all generated data or specific parameters such as total cells and PI-positive cells. Click “Export” to generate and save an Excel (.xlsx) file on your computer (Figure 26).
94. If you need to view or export acquired and analyzed images, switch to “Plate View,” review the images, and export as necessary.
95. Exported data can be processed and presented using software such as GraphPad Prism or your preferred analysis software (Figure 27).

***Note:*** Proper software recognition of the microfluidic chip layout is crucial for accurate imaging and analysis. Ensure channels on the chip are correctly identified and imaged within the software.

## Expected outcomes

This protocol details methodologies for fabricating microfluidic devices and acquiring, culturing, and preparing pulmonary arterial endothelial cells (PAH-ECs) for seeding onto these devices. Utilizing an image-based cellular cytometer, cells seeded on the microfluidic chip are counted efficiently. Typically, PAH-ECs cultured at early passages reach confluence within four days. Live and dead cell populations are distinguished through labeling with Hoechst dye and propidium iodide, respectively. By providing comprehensive instructions for device manufacturing, cell culture, and on-chip cell counting, this protocol supports PAH research in evaluating cell morphology, conducting drug screenings, and assessing therapeutic interventions.

The microfluidic devices described here are not reusable due to concerns regarding cross-contamination and the potential loss of surface functionalization after initial use. However, the fabrication process is highly scalable and has been described in detail in our previously published protocol. Briefly, a single mold can cast up to six devices, and a single user can easily handle four molds to prepare approximately 20 chips with minimal effort. We published detail protocol previously on how fabrications can be done.^3^^,^^5^ To increase experimental throughput, six microfluidic chips can be simultaneously placed into a standard 6-well plate, enabling batch imaging using an automated cytometry protocol. Imaging of all six chips typically requires 30–45 min for completion, depending on resolution and scan settings. These scalable fabrication and parallel imaging strategies make the protocol suitable for medium- to high-throughput analysis of PAH-EC samples in drug screening, cell viability studies, and therapeutic testing. For representative data and detailed experimental outcomes, please refer to our previously published Study.^1^

## Quantification and statistical analysis

Cell count data were plotted using GraphPad Prism (version 10.1.2, GraphPad Software LLC, Boston, MA). The sample size (n) reflects the number of independent chip imaging repetitions performed using the cellular image cytometer.

## Limitations

A primary limitation of this protocol is its dependence on the Pulmonary Hypertension Breakthrough Initiative (PHBI) as the exclusive provider of pulmonary arterial hypertension (PAH) cells. Relying on a single biobank may restrict generalizability, since donor variability, sample handling, and processing methods can influence cell phenotype and quality. Furthermore, the number of PHBI-derived cells available for distribution is limited, which may pose challenges for scalability and reproducibility across laboratories. Similar considerations have been noted in earlier STAR Protocols reports.^2^

## Troubleshooting

### Problem 1

Cell Migration or Leakage Between Channels During Seeding in a Multichannel Microfluidic Chip (Steps 43–45, 58).

### Potential solution

Evaluate the chip for inter-channel leakage during the poly-D-lysine (PDL) coating step. Following PDL coating and thorough washing, incubate the chip at 80°C for a minimum of 24 h and up to 72 h. This step is critical because the PDL-coated surface requires at least 24 h to partially restore its hydrophobicity, which helps retain the cell suspension within its designated channel and prevents leakage into adjacent layers during seeding. Additionally, best practice is to fabricate microfluidic chips in a clean room environment, as dust particles or microscopic debris can compromise channel integrity and lead to leakage. Ensuring a dust-free fabrication process improves reproducibility and performance of the device.

### Problem 2

Maintaining proper humidity is critical for successful cell growth in microfluidic chips. Standard humidity levels in cell culture incubators are often insufficient, leading to evaporation of media and compromised cell viability during long-term culture at 37°C (Step 56).

### Potential solution

To prevent media evaporation and maintain optimal humidity, place the microfluidic chips inside a sterile, empty pipette tip box filled with sterile water, as shown in Figure 3. This setup creates a localized humidified chamber, enhancing environmental stability and supporting effective cell growth within the chip during incubation.

### Problem 3

Weak or inconsistent cell attachment in gelatin-coated T-flasks, 6-well, or 24-well plates during initial culture and expansion can hinder cell viability and proliferation (Step 62).

### Potential solution

To enhance cell adherence, increase the gelatin concentration to up to 0.5% during the coating process. A higher gelatin concentration provides a denser and more adhesive surface matrix, which can significantly improve initial cell attachment, particularly for primary pulmonary arterial cells.

### Problem 4

Cracking of the glass slide during edge trimming, prior to placement inside a 6-well plate, can compromise the structural integrity of the microfluidic chip and adversely affect downstream imaging (Step 69).

### Potential solution

This step must be performed with extreme caution, as damage to the glass can interfere with imaging and chip function. To minimize the risk of cracking, use a very sharp, high-quality diamond cutter and apply gentle, controlled pressure while scoring the glass edges. Carefully trim only the excess glass necessary for fitting the chip into the well plate. Even if a minor crack occurs, imaging may still be possible depending on the location and severity of the damage, so it is worth testing before discarding the chip.

### Problem 5

Cells may enter a senescent-like state and exhibit slow or halted proliferation if they become overly confluent. This contact-induced growth inhibition can impair subsequent passaging and experimental outcomes (Step 22).

### Potential solution

Monitor cell confluency daily and passage the cells when they reach no more than 70% confluency. Allowing cells to reach full confluence can trigger contact inhibition, leading to reduced proliferation and viability in subsequent passages. Maintaining cells in the exponential growth phase supports healthy expansion and consistent performance across experiments. Additional details on our standard cell culture techniques, previously applied to cancer and other cell models, are available in our earlier publication.^6^^,^^7^^,^^8^^,^^9^^,^^10^^,^^11^^,^^12^^,^^13^

## Resource availability

### Lead contact

Further information and requests for resources and reagents should be directed to and will be fulfilled by the lead contact, Fakhrul Ahsan (fakhrul.ahsan@cnsu.edu).

### Technical contact

Technical questions on executing this protocol should be directed to and will be answered by the technical contact, Md Ibrahim (md.ibrahim@cnsu.edu).

### Materials availability

This study did not generate new unique reagents.

### Data and code availability

Added data are available from the corresponding author on request. There is no code generated during this study.

## Acknowledgments

This work was supported by NIH grants R01HL144590 and R43HL169134 and the Cardiovascular Medical Research and Education Fund. The graphical abstract was created in BioRender. Ibrahim, M. (2025) https://BioRender.com/gyf2sto.

## Author contributions

Conceptualization, M.I. and F.A.; methodology, M.I., S.M.M., T.S., and F.A.; investigation, M.I. and S.M.M.; writing – original draft, M.I. and S.M.M.; writing – review and editing, M.I., T.N., E.N., T.L., L.C., M.A.S., K.R.S., and M.S.H.; validation, M.I.; visualization, M.I.; funding acquisition, F.A.; resources, T.S. and A.E.-S.; supervision, F.A.; project administration, F.A.

## Declaration of interests

F.A. discloses partial ownership of Medludics LLC, located in Elk Grove, California. M.I. discloses partial ownership of Oncovask Therapeutics LLC in Sacramento, California. The contents reported/presented within do not represent the views of the Department of Veterans Affairs or the United States Government.

## Declaration of generative AI and AI-assisted technologies in the writing process

During the preparation of this work, the authors used ChatGPT (GPT-4o/5) to proofread and increase readability and clarity. After using this tool, the authors reviewed and edited the content as needed and took full responsibility for the content of the publication.
